# Review: Systemic inflammation after stroke. Therapy and perspective

**DOI:** 10.1007/s11357-025-02070-1

**Published:** 2026-01-07

**Authors:** Abuzan Mihaela, Andreea Cercel, Thorsten R. Doeppner, Dirk M. Hermann, Roxana Surugiu, Denisa F. V. Pirscoveanu, Aurel Popa-Wagner

**Affiliations:** 1 Department of Neurology, University Medical Center Göttingen, 37075 Göttingen, Germany; 2 Department of Neurology, University of Giessen Medical School, 35392 Giessen, Germany; 3 Chair of Vascular Neurology and Dementia, Department of Neurology, University Hospital Essen, 45147 Essen, Germany; 4 Department of Neurology, University of Medicine and Pharmacy Craiova, Craiova, Romania; 5 Experimental Research Center for Normal and Pathological Aging, University of Medicine and Pharmacy Craiova, Craiova, Romania; 6 Doctoral School, University of Medicine and Pharmacy, Craiova, Romania

**Keywords:** Ischemic stroke, Systemic inflammation, Aging, Obesity, Anti-inflammatory therapy, Rehabilitation

## Abstract

Systemic inflammation following ischemic stroke is driven by a complex interplay among pro-inflammatory cytokines, immune cell activation, and neurovascular dysfunction. Both aging and obesity significantly amplify this inflammatory response, exacerbating stroke severity and impeding recovery. Aging induces a chronic low-grade inflammatory state—referred to as *inflammaging*—that heightens vulnerability to stroke-induced brain injury. Similarly, obesity promotes a persistent pro-inflammatory milieu that disrupts metabolic and immune homeostasis, further worsening neurological outcomes. The combined effects of aging and obesity pose a substantial barrier to effective stroke rehabilitation and long-term recovery. To improve post-stroke care, future research should focus on three key areas. First, there is a pressing need for targeted therapies that modulate systemic inflammation with minimal side effects. Anti-inflammatory agents such as minocycline have shown promise in preclinical models, but clinical validation is needed. Second, elucidating the molecular mechanisms linking aging, obesity, and systemic inflammation—such as the roles of adipokines and immune cell phenotypes—may reveal novel therapeutic targets. Finally, personalized treatment strategies that consider individual risk factors like age and obesity are essential to optimize stroke management and rehabilitation. Given the limited efficacy of current stroke treatments, prioritizing prevention by identifying high-risk individuals is critical. Recognizing non-modifiable risk factors can support more intensive intervention on modifiable ones and guide vigilance toward vulnerable populations. Overall, advancing our understanding of systemic inflammation and its modifiers will be key to developing innovative, patient-specific therapies aimed at improving outcomes and quality of life for stroke survivors.

## Background

Stroke is an important health issue caused by an impairment in blood flow which ends in the loss of neurons [1]. Every year, 15 million people worldwide attend emergency departments due to strokes, of which 5.5 million die and more than 5 million become permanently disabled. The two main types of stroke are ischemic stroke (approximate 87%) and hemorrhagic stroke (around 13%, including subarachnoid hemorrhages) [2].

Stroke has a high rate of mortality and is the second most common cause of death worldwide [3]. After the age of 55, the risk of stroke doubles every ten years. Over the next twenty years, there will be an ongoing increase in the prevalence of stroke due to an aging population [3]. In part due to the significant increase in the global aging population, ischemic stroke incidence is rising. Furthermore, age is a risk factor for cardiovascular and cerebrovascular events that share inflammatory pathophysiological mechanisms due to the prevalence of multiple comorbidities, including obesity, heart disease, atherosclerosis, hypertension, and diabetes mellitus. These factors may even aggravate or increase the development of other damaging medical conditions and worsen the long-term outcome [4].

Multi-morbidity is common among older adults and highly prevalent in stroke patients—affecting 89% of those aged 65 and older, and 60% of those under 65. Stroke survivors may develop additional comorbidities over time, potentially interacting with cardiovascular risk factors to alter stroke risk. These comorbidities significantly impact hospital readmission rates, functional recovery, and mortality [5].

Currently, the only available methods of treating ischemic stroke are endovascular therapy (catheter-based mechanical thrombectomy) or acute revascularization techniques, which involve the administration of thrombolytic medication [6]. Systemic inflammation represents one of the biological processes that have recently come into focus as important pathophysiological processes of stroke, but it has received less attention than these acute neuroprotective strategies. One significant factor influencing both the short- and long-term outcomes of stroke patients has been reported to be poststroke inflammation, whose effects are greatly amplified by advanced aging and the prevalence of multiple comorbidities [7].

Age is one of non-modifiable risk factors; obesity is a modifiable risk factor. These factors affect not only the incidence of stroke but also the severity of systemic inflammation, which is a key aspect of post-stroke pathology [8]. Therefore, understanding and addressing systemic inflammation post-ischemic stroke is crucial for developing new diagnostic, prognostic, and therapeutic strategies that can modulate the immune response and improve the long-term functional and cognitive outcomes of stroke survivors.

### Chronic low-grade systematic inflammation in aging

Aging is accompanied not only by a gradual decline in physiological and regenerative capacities but also by the emergence of a chronic, low‐grade, systemic inflammatory state, commonly referred to as “inflammaging” [9]. This persistent, subclinical inflammation is now widely recognized as a key driver of aging and a common denominator in the pathogenesis of multiple age‐associated disorders, including cardiovascular disease, type 2 diabetes, neurodegenerative conditions such as Alzheimer’s and Parkinson’s disease, sarcopenia, and several types of cancer [10, 11].

The underlying mechanisms of inflammaging are multifactorial and stem from a dynamic and cumulative interaction between intrinsic and extrinsic aging processes. One major contributor is immunosenescence, the gradual remodeling and dysfunction of the immune system with age, which leads to impaired resolution of inflammatory responses and a proinflammatory immune phenotype characterized by increased production of cytokines such as IL-6, TNF-α, and CRP [12]. Additionally, metabolic dysregulation—including insulin resistance, mitochondrial dysfunction, and the accumulation of senescent cells—further exacerbates inflammatory signaling via the activation of inflammasomes and the senescence-associated secretory phenotype (SASP) [13, 14].

Alterations in the gut microbiome—marked by reduced microbial diversity and an increase in pro-inflammatory taxa—have also been implicated in promoting systemic inflammation through increased intestinal permeability (“leaky gut”) and translocation of microbial components such as lipopolysaccharide (LPS) into the circulation [15, 16]. Chronic viral and bacterial infections, including cytomegalovirus (CMV), can induce sustained immune activation and skew T-cell populations toward an exhausted phenotype, further fueling inflammaging [17]. Finally, environmental and lifestyle factors—such as poor diet, physical inactivity, psychosocial stress, and exposure to pollutants—act as both triggers and amplifiers of chronic inflammation in older adults [11, 18].

Given its systemic nature and multifactorial origins, inflammaging represents a crucial therapeutic target in aging research. Strategies aimed at modulating immune function, reducing senescent cell burden, restoring gut microbiota composition, and promoting anti-inflammatory lifestyles are currently being explored to mitigate the deleterious effects of chronic inflammation and extend healthspan [19, 20].

## Inflammasome activation in aging

Senescent cells exhibit a complex pro-inflammatory response known as the senescence-associated secretory phenotype (SASP). Mechanistically, SASP expression is regulated by inflammasome-mediated IL-1 signaling. In senescent cells, the inflammasome and IL-1 signaling pathways are activated, and IL-1α expression alone can induce SASP activation, reinforcing the senescent state [21].

More recently it has been shown that the NLRP3 inflammasome regulates systemic low-grade, age-associated activation of the noncanonical caspase-11 inflammasome. Notably, its ablation protected mice from age-related increases in innate immune activation, CNS transcriptomic alterations, and astrogliosis. Furthermore, IL-1 signaling mediated NLRP3 inflammasome-dependent enhancements in cognitive function and motor performance in aged mice [22]. Another study has shown that ablation of NLRP3-inflammasome protected mice from age-related increased insulin sensitivity, reduced IGF-1 and leptin/adiponectin ratio levels, and reduced cardiac damage probably by an inhibition of the PI3K/AKT/mTOR pathway and increased autophagy and increased SIRT1 protein expression [23].

## Age-related disbalance of the immune system

As we age, the immune system undergoes significant changes. The aging immune system experiences a paradoxical shift: while the adaptive immune response typically declines, innate immune responses may become overactive, leading to a dysregulated immune state. The reduced efficiency of the adaptive immune system, particularly the decline in naïve T and B cell populations, weakens the body’s ability to mount specific responses to new infections and efficiently clear damaged cells. Meanwhile, heightened innate immune activity, characterized by increased pro-inflammatory cytokine production, can exacerbate tissue damage and hinder proper immune resolution. This imbalance contributes to *inflammaging*, a chronic, low-grade inflammatory state implicated in various age-related diseases, including neurodegenerative disorders, cardiovascular diseases, and metabolic dysfunction.

This underscores the importance of developing therapeutic strategies, such as senolytics and immune-modulating interventions, to restore immune balance and mitigate the detrimental effects of chronic inflammation in aging individuals [19]. have demonstrated that senescent cell clearance improves several domains of age-related tissue decline including central nervous system dysfunction.

## Senescence‐associated secretory phenotype

With age, most cells enter a state of permanent cell cycle arrest mainly coordinated by p16^INK4a^ and p21^Waf1/Cip1^, gradually adopting a senescent phenotype due to a myriad of factors. Although these cells no longer divide, they remain metabolically active and secrete a variety of pro‐inflammatory cytokines, chemokines, growth factors, and proteases—collectively known as the senescence‐associated secretory phenotype (SASP). The accumulation of senescent cells is another key factor that in conjuction with the dysregulation of the immune system aggravates systemic inflammation and tissue dysfunction [24, 25].

Recent studies have begun to explore how the cyclic GMP-AMP synthase (cGAS)–STING pathway, a key sensor of cytosolic DNA, is activated in senescent cells [26]. The activation of this pathway appears to contribute to the secretion of inflammatory mediators during aging. Indeed, blockade of STING suppresses the inflammatory phenotypes of senescent human cells and tissues, attenuates aging-related inflammation in multiple peripheral organs and the brain in mice, and leads to an improvement in tissue function.

Innate immunity within the central nervous system (CNS) is primarily maintained by resident microglia. These cells play a crucial role in immune surveillance and facilitate coordinated interactions between the immune system and the brain. However, with normal aging, microglia develop a hypersensitive and pro-inflammatory phenotype, known as priming [27], particularly in the context of aging and neurodegeneration.

As aging progresses, primed microglia exacerbate neuroinflammation, thereby altering CNS function [27, 28]. Thus, it has been shown that activation of STING triggers reactive microglial transcriptional states, neurodegeneration, and cognitive decline. Cytosolic DNA released from perturbed mitochondria elicits cGAS activity in aged microglia, defining a mechanism by which cGAS–STING signaling is engaged in the aging brain.

Single-nucleus RNA sequencing (snRNA-seq) analysis of microglia and hippocampi from a cGAS gain-of-function model revealed that senescent cells accumulate in both the brain and spinal cord with age. This was confirmed using p16 reporter (p16-luc) mice, which are increasingly used to visualize senescent cells in vivo. Subsequent single-cell RNA sequencing (scRNA-seq) and detailed histological analysis revealed that senescent cells were primarily detected in microglia within CNS white matter regions. Furthermore, knockout of p16INK4a, a key senescence inducer, attenuated the neuroinflammatory phenotype [29–31].

## Vascular wall and the SASP

Recent studies have highlighted the significant role of the SASP in the development and progression of cardiovascular diseases, particularly through its impact on large blood vessels. Therefore, understanding the mechanisms by which SASP influences vascular health opens new avenues for therapeutic intervention. Targeting senescent cells or modulating their secretory profiles may provide promising strategies to mitigate vascular aging and reduce the burden of cardiovascular diseases [32].

There are two main pillars responsible for atherosclerotic lesion formation: (i) endothelial dysfunction and (ii) lipid retention and oxidative modification within the arterial vessel wall. Persistent inflammation activates VSMCs to migrate toward the lesion site, where a portion of VSMCs transdifferentiate to alternative cell lineages of macrophage-like and mesenchymal stem cell-like phenotype and another portion become senescent [33, 34]. acquiring a SASP that involves production of metalloproteinases (MMPs) and ECM remodeling, responsible for pathological intimal thickening and fibrous plaque formation. Recent studies have outlined the contribution of the SASP to the pathophysiology of cardiovascular diseases by promoting inflammation and tissue remodeling [35]. In atherosclerosis, for instance, SASP factors not only exacerbate local inflammation but also induce senescence in neighboring healthy cells, further amplifying vascular dysfunction [36]. Similarly, the accumulation of senescent cells within the vascular endothelium and smooth muscle layers promotes plaque formation and instability. Senescent vascular smooth muscle cells (VSMCs) exhibit a SASP characterized by increased production of interleukin-1α (IL-1α), which fosters chronic inflammation within atherosclerotic plaques. This inflammatory environment accelerates plaque progression and instability, thereby increasing the risk of adverse cardiovascular events [36].

A growing body of clinical and experimental studies highlights that the accumulation of senescent cells in the cardiovascular system is associated with poor outcomes in heart failure and myocardial infarction. The SASP factors secreted by these cells can exacerbate tissue damage and impair repair mechanisms, ultimately leading to worsened cardiac function [35, 37, 38].

## Age-related neuroinflammation and the SASP

Recent studies have shed light on the intricate relationship between the SASP, large blood vessel pathology, and age-related neuroinflammation. While SASP can have beneficial roles, such as tumor suppression the influence of SASP extends to the central nervous system. Senescent cells accumulate in the aging brain and secrete SASP factors that exacerbate neuroinflammation. This heightened inflammatory state contributes to synaptic loss, neurodegeneration, and cognitive decline, which are hallmark features of neurodegenerative diseases such as Alzheimer’s disease [39].

The role of the SASP in triggering neuroinflammation has been investigated using transgenic mice, which enable the systemic removal of senescent cells. These mice are engineered to allow the inducible elimination of p16Ink4a-expressing senescent cells, typically through the administration of AP20187, which triggers apoptosis in the targeted cells. This model establishes a causal link between cellular senescence and SASP-driven pathology, providing insights into how senescent cells contribute to tissue dysfunction, chronic inflammation, and aging-related diseases, including neurodegeneration and stroke.

Using this mouse model, studies have shown that p16-positive myeloid cells—exhibiting both senescent and disease-associated activation signatures, including the upregulation of chemoattractant factors—accumulate in the aged mouse brain. Additionally, senescent brain myeloid cells and infiltrating immune cells increase with age. However, their numbers are partially restored to youthful levels through the clearance of p16-positive senescent cells in female p16-InkAttac mice, a process associated with the preservation of cognitive function [40].

A decline in mitochondrial energy production is another significant factor contributing to microglial activation and subsequent neuroinflammation. Research has shown that impaired oxidative phosphorylation (OXPHOS) and a shift towards glycolysis in dysfunctional microglia exacerbate neuroinflammatory responses [41]. For example, a study has demonstrated that a breakdown in metabolic reprogramming leads to microglial dysfunction in Alzheimer’s disease, highlighting the role of mitochondrial dysfunction in promoting a pro-inflammatory phenotype characterized by elevated levels of inflammatory cytokines [42]. Additionally, dysfunctional mitochondria resulting from mitochondrial DNA (mtDNA) damage and impaired mitophagy further contribute to microglial activation in the central nervous system [43].

### Vacular aging and obesity, risk factors for ischematic stroke

Vascular aging is marked by inflammation, endothelial dysfunction, and the progressive stiffening of arteries. It is also linked to heightened production of mitochondrial reactive oxygen species, reduced nitric oxide availability, and the uncoupling of endothelial nitric oxide synthase [44].

Promoting angiogenesis—the growth of microvascular structures—would be beneficial for stroke. Proliferation, migration, and recruitment of endothelial cells are characteristic features of capillary sprouting during angiogenesis. There have been studies that aging impairs each of these dynamics. Endothelial senescence has been related to impaired angiogenesis, and alterations in growth factor signaling and the extracellular matrix provide additional data for decreased endothelial cell dynamics in elderly circumstances. In terms of growth factors, there is a reduction in the expression of platelet-derived growth factor (PDGF) and vascular endothelial growth factor (VEGF), and there is a decrease in the phosphorylation of the receptors, which decreases the response to basic fibroblast growth factor (bFGF). Regarding the extracellular matrix, it has been demonstrated that the matrix metalloproteinases (MMPs) that weaken it have increased, while MMP inhibitor levels have apparently decreased [45].

## Obesity as a risk factor

Over the past 50 years, there has been a significant increase in the obesity rate worldwide. A person is considered obese if their body mass index (BMI) is greater than or equal to 30. Overweight people have a BMI between 25 and 29.9 [46].

Obesity is a complex disease with genetic and behavioral causes. Patients may decrease their risk of stroke and ASCVD by controlling their obesity using medication and lifestyle modifications. Weight loss indirectly contributes by lowering blood pressure and cholesterol, two risk factors. More recently, the association between metabolic health and BMI and the risk of stroke and cardiovascular disease has been studied. Generally speaking, ischemic stroke risk is higher in metabolically unhealthy obese people than in metabolically healthy persons with a normal BMI [47].

Metabolic syndrome, significantly influenced by obesity, is a cluster of cardiovascular risk factors that greatly heightens the likelihood of stroke. It is defined by the presence of any three of the following conditions: abdominal obesity, elevated triglycerides, reduced HDL cholesterol, prehypertension or hypertension, and insulin resistance or diabetes. This syndrome fosters a proinflammatory and prothrombotic state, marked by decreased levels of antioxidants and increased concentrations of C-reactive protein, fibrinogen, and plasminogen activator inhibitor-1. Consequently, individuals with metabolic syndrome are at a considerably higher risk of experiencing ischemic stroke [48].

**Fig. 1:**
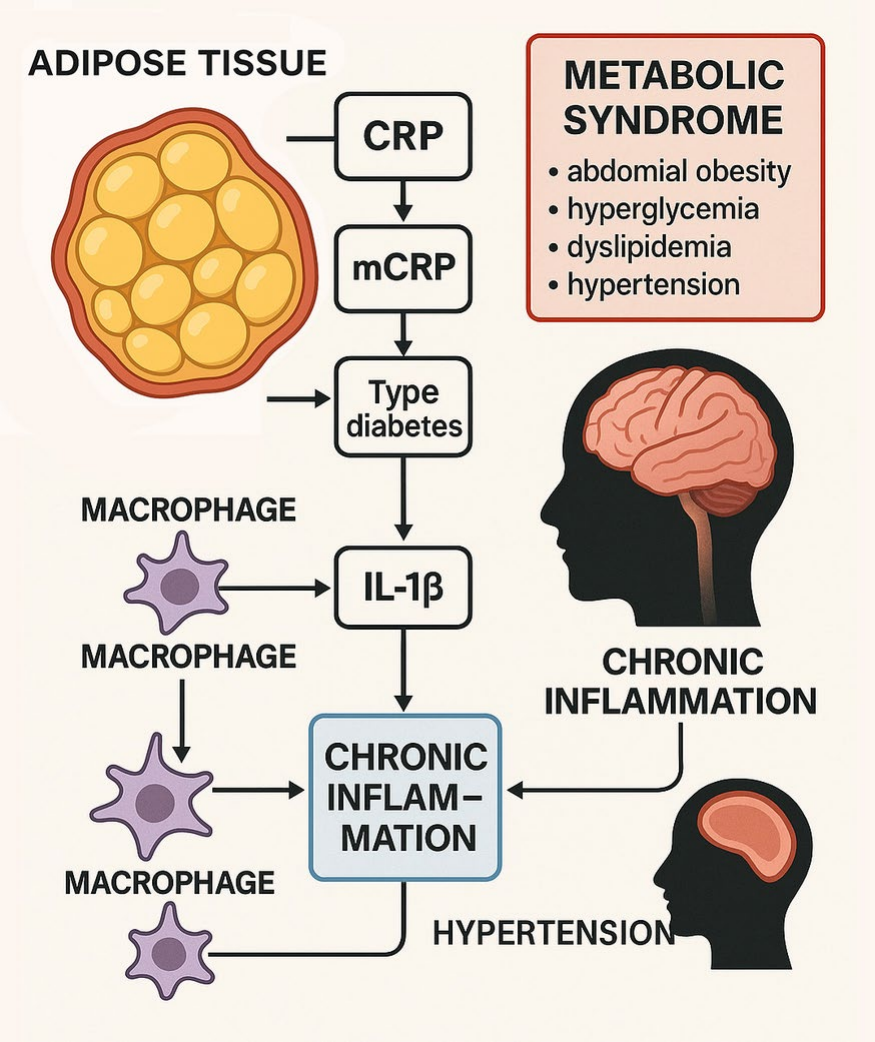
Molecular pathways linking obesity-induced inflammation to stroke risk. Obesity promotes macrophage activation in adipose tissue, leading to the release of pro-inflammatory cytokines such as interleukin-1β (IL-1β), IL-6, and tumor necrosis factor-α (TNF-α). These mediators contribute to systemic chronic inflammation, oxidative stress, and endothelial dysfunction. Elevated levels of C-reactive protein (CRP) and its monomeric form (mCRP)—generated at sites of oxidative and vascular injury—amplify inflammation and microvascular damage, promoting platelet adhesion and barrier disruption. A lower CRP:mCRP ratio reflects chronic vascular inflammation and endothelial injury, while a higher ratio indicates an acute-phase response. These inflammatory and metabolic disturbances drive insulin resistance, non-alcoholic fatty liver disease, and hypertension, collectively heightening the risk of ischemic stroke

Beyond conventional systemic markers of inflammation, recent studies underscore the importance of monomeric C-reactive protein (mCRP) as a clinically relevant indicator of vascular pathology in metabolic and cerebrovascular disease. Generated from the dissociation of native pentameric CRP at sites of oxidative stress and endothelial damage, mCRP displays pronounced pro-inflammatory, pro-thrombotic, and endothelial-activating effects. It accumulates in atherosclerotic and ischemic vascular lesions, where it promotes leukocyte recruitment, platelet adhesion, and microvascular dysfunction. Clinically, lower CRP:mCRP ratios have been linked to chronic vascular inflammation and endothelial injury, whereas higher ratios reflect acute-phase inflammatory responses. As such, assessing mCRP levels or CRP:mCRP ratios may improve risk stratification for endothelial dysfunction, blood–brain barrier disruption, and recurrent ischemic events, particularly in older or obese individuals with metabolic syndrome [49–56]. (Fig. 1).

Obesity-induced inflammation plays a critical role in increasing the risk of stroke by contributing to related metabolic diseases such as type 2 diabetes, fatty liver, and hypertension. In obesity, macrophages in adipose tissue trigger systemic chronic inflammation, which disrupts the function and metabolism of various cells, worsening these conditions. Inflammatory factors like interleukin-1β (IL-1β) released by macrophages are particularly important in promoting insulin resistance and metabolic dysfunction, making obesity-induced inflammation a significant risk factor for stroke [57].

## The interplay between aging and obesity

The interplay between aging and obesity significantly amplifies the risk of ischemic stroke. As people age, their chances of developing obesity increase due to factors like a slower metabolism and reduced physical activity. This combination of age-related vascular changes and obesity-induced inflammation exacerbates stroke risk. Both aging and obesity contribute to a heightened inflammatory state, with elderly obese individuals experiencing an intensified systemic inflammatory response after a stroke, worsening their prognosis. This population is also more likely to have multiple comorbidities, complicating management and increasing the risk of recurrent stroke [5, 58, 59].

## Metabolic dysfunction and adipose tissue inflammation

Metabolic changes are a hallmark of aging, contributing to systemic metabolic disturbances, including impaired insulin signaling and disrupted glucose homeostasis. Insulin resistance and metabolic syndrome, both common in aging, further contribute to inflammation by altering cellular energy metabolism and promoting oxidative stress. This metabolic dysregulation is both a cause and a consequence of chronic inflammation, creating a feedback loop that accelerates tissue aging and disease progression.

The redistribution of adipose tissue, particularly the increase in visceral fat, is closely linked to metabolic dysfunction. Adipocytes and infiltrating immune cells within adipose tissue secrete various pro-inflammatory adipokines, such as interleukin-6 (IL-6) and tumor necrosis factor-alpha (TNF-α), which exacerbate systemic inflammation [60]. A key early study demonstrated that obesity significantly increases macrophage infiltration in adipose tissue, correlating with elevated expression of pro-inflammatory cytokines such as TNF-α and IL-6 [61]. These cytokines not only disrupt insulin signaling but also promote chronic inflammation, further exacerbating metabolic dysfunction. Additionally, research on adipose tissue heterogeneity suggests that visceral adipose tissue (VAT) is more prone to inflammation than subcutaneous fat, making it a critical target for metabolic disease interventions [62]. Chronic low-grade inflammation in adipose tissue is driven by increased infiltration of pro-inflammatory macrophages, dysregulated adipokine secretion, and activation of inflammatory signaling pathways such as NF-κB and JNK [63].

### Dysbiosis, gut barrier function, and systematic inflammation

The gut microbiota is essential for maintaining intestinal homeostasis and systemic immune balance. Dysbiosis—an imbalance in microbial composition and function—has been linked to various pathological conditions, including neuroinflammatory and metabolic disorders. A key consequence of dysbiosis is the disruption of gut barrier integrity, resulting in increased intestinal permeability (“leaky gut”) and systemic inflammation [64].

Microbial diversity plays a critical role in preserving the gut barrier. Beneficial commensals such as *Lactobacillus* and *Bifidobacterium* produce short-chain fatty acids (SCFAs), especially butyrate, which support epithelial integrity by enhancing tight junction protein expression [65, 66]. In contrast, depletion of these microbes—along with overgrowth of opportunistic pathogens like *Escherichia coli* and *Clostridium difficile*—disrupts epithelial tight junctions and increases paracellular permeability [67]. These changes activate inflammatory pathways and trigger systemic immune responses.

Microbial metabolites also directly modulate barrier function. Lipopolysaccharides (LPS) from Gram-negative bacteria, for instance, stimulate Toll-like receptor 4 (TLR4), promoting the release of pro-inflammatory cytokines and further weakening tight junctions [68]. Animal models have shown that this barrier dysfunction facilitates the translocation of bacterial products into circulation, contributing to behavioral and physiological changes consistent with depression [69].

The systemic spread of microbial components, including LPS and peptidoglycans, contributes to chronic low-grade inflammation—a hallmark of metabolic syndrome, neurodegeneration, and cardiovascular disease. A recent meta-analysis confirmed that individuals with metabolic syndrome exhibit elevated serum LPS levels, correlating with increased C-reactive protein (CRP) and pro-inflammatory cytokines such as tumor necrosis factor-alpha (TNF-α) and interleukin-6 (IL-6) [63]. In the brain, dysbiosis-driven inflammation exacerbates neuroinflammation by activating microglia and impairing blood–brain barrier (BBB) integrity [70].

Clinical studies further support the role of gut barrier dysfunction in disease progression. In inflammatory bowel disease (IBD), elevated serum levels of barrier-disruption markers like zonulin and claudin-2 are associated with disease severity and systemic inflammation [71]. Similarly, in type 2 diabetes, increased gut permeability correlates with insulin resistance and inflammation, highlighting the significance of the gut-liver axis in metabolic dysregulation [72]. Therefore, restoring microbial balance is increasingly recognized as a promising approach to strengthen gut barrier function and mitigate systemic inflammation and inflammaging.

### Lifestyle factors and systematic inflammation

Lifestyle choices play a critical role in modulating the inflammatory landscape in aging. Diets high in processed foods, refined sugars, and saturated fats have been associated with increased inflammation. Conversely, adherence to anti-inflammatory diets—such as the Mediterranean diet—has been shown to lower inflammatory markers [73]. Regular physical activity, stress management, and avoidance of tobacco smoke and environmental pollutants are likewise essential for mitigating systemic inflammation.

## Diet and inflammation

It has been hypothesized that metabolic inflammation may be driven by a range of adverse dietary factors, including saturated fatty acids and certain sugars, suggesting that specific dietary triggers may be particularly relevant beyond simple excessive dietary intake, which often manifests as obesity [74, 75].

A multicenter trial in Spain involving individuals at high cardiovascular risk compared the effects of three different diets: a Mediterranean diet supplemented with extra-virgin olive oil, a Mediterranean diet supplemented with mixed nuts, and a control diet. The study reported that cardiovascular events were lower among those assigned to a Mediterranean diet supplemented with extra-virgin olive oil or nuts than among those following a reduced-fat diet [74].

The Dietary Inflammatory Index (DII) was recently developed to estimate the inflammatory potential of different dietary patterns. Although the DII has been suggested to be associated with metabolic syndrome and other cardio-metabolic diseases, the precise nature of this relationship remains unclear. While the DII score appears to be a useful tool for studying the inflammatory capacity of diet and understanding the relationships between diet, inflammation, and cardio-metabolic diseases, a causative role has not yet been clearly demonstrated [76, 77].

Physical activity has also been linked to systemic inflammation. A study aimed at determining whether higher levels of physical activity and reduced sedentary behavior are associated with lower levels of inflammation, as indicated by inflammatory and hemostatic biomarkers, in older men found that moderate to vigorous physical activity benefits general health by lowering serum inflammatory markers [78].

Other less-studied factors impacting systemic inflammation include sleep quality and social stress. Sleep is reciprocally linked to the innate immune system; substantial evidence from translational animal models has revealed that inflammatory cytokines homeostatically regulate sleep [79]. The homeostatic role of sleep in regulating inflammatory biology dynamics, as well as the impact of sleep disturbances and extreme sleep durations on the innate immune system at systemic, cellular, and genomic levels, has implications for inflammaging and molecular processes of aging [80].

In modern times, chronic social threats can drive the development of sleep disturbances in humans by acting via the HPA axis and ultimately affecting cortisol levels. This, in turn, can contribute to the dysregulation of inflammatory responses [81, 82]. A cross-sectional study investigated the association between psychological distress and inflammatory proteins in a young, healthy, representative population of English adults. The findings suggested that psychological distress may negatively impact inflammatory processes in young adulthood before the onset of chronic health conditions such as hypertension and cardiovascular disease [83].

Despite the growing body of research in this field, large, longitudinal, well-controlled studies are required to move beyond associations and establish a causal link between psychosocial stress and systemic inflammation.

### Post-stroke systematic inflammation. Role of aging and obesity

Systemic post-stroke inflammation plays a critical role in determining both immediate and long-term outcomes for stroke patients. Aging and obesity not only increase systemic inflammation but also contribute to a range of comorbidities including heart disease, atherosclerosis, hypertension, and diabetes mellitus that share inflammatory mechanisms with stroke. Furthermore, inflammation associated with aging and obesity can worsen pre-existing conditions and lead to additional issues such as post-stroke infections and long-term vascular dementia, ultimately impairing stroke recovery and outcomes [84].

Stroke injury releases immune active molecules (DAMPs), which activate local immune cells (microglia and astrocytes) and recruit circulating immune cells to the brain [85]. After a cerebrovascular event, macrophages and activated microglia migrate into the lesion site to clear debris from apoptotic neurons, a process typically protective. However, in aging brains, this process can become detrimental. Aged macrophages may produce prostaglandin E2, which inhibits T-cell growth, and CCR6, which attracts more macrophages, further disrupting cytokine balance [86]. Increased systemic inflammatory cytokines can alter macrophage behavior, leading to neuronal damage and impaired synaptic plasticity. Astrocytes contribute by releasing cytokines, chemokines, and reactive oxygen species (ROS), creating a feedback loop with microglia that sustains inflammation [87].

Microglia release pro-inflammatory cytokines such as IL-1β, TNF-α, and IL-6, recruiting peripheral immune cells to the brain and intensifying inflammation [88]. The compromised blood–brain barrier (BBB) permits leukocytes like neutrophils, monocytes, and T-cells to infiltrate the brain, further exacerbating damage. This localized brain inflammation triggers systemic cytokine release, leading to a broader inflammatory response that can worsen stroke outcomes [89, 90].

Neutrophils play a crucial role in host defense by performing functions such as enzyme secretion, phagocytosis, cytokine production, and the generation of reactive oxygen species (ROS) and neutrophil extracellular traps (NETs). Their ability to clear cellular debris is essential for an effective anti-inflammatory response. However, in aged hosts, neutrophils may exhibit altered phagocytosis, excessive NET release, and increased ROS production. These changes can impair the body’s response to infections or sterile injuries, exacerbating chronic inflammation and potentially damaging healthy tissues [91]. Additionally, dendritic cells, essential for antigen presentation and immune regulation, exhibit reduced ability to stimulate CD4 + and CD8 + T-cells and promote interferon-γ secretion with age [92, 93].

## Impact of obesity on post-stroke systemic inflammation

Obesity, a major public health issue and the second leading preventable cause of death after tobacco, has seen its prevalence double since the 1980s. Inflammation and immune mechanisms are critical in stroke risk and outcomes. Traditionally linked to disruptions in lipid and glucose metabolism, obesity is now recognized for its inflammatory component. Obesity measures such as BMI correlate positively with inflammation markers including C-reactive protein (CRP), interleukin-6 (IL-6), tumor necrosis factor alpha (TNFα), monocyte chemoattractant protein 1 (MCP-1), interleukin-8 (IL-8), and soluble intracellular adhesion molecule 1 (ICAM-1), supporting the view of obesity as an inflammatory condition [94, 95].

Adipose tissue plays a crucial endocrine role through adipokines, such as leptin and adiponectin. These adipose-derived proteins not only regulate immune functions and energy balance but also influence various physiological processes, including blood clotting, lipid and glucose metabolism, blood pressure, insulin sensitivity, and angiogenesis [96].

In healthy adults, plasma adiponectin levels are high, but they decrease with increasing adiposity. Adiponectin has notable anti-inflammatory and neuroprotective properties, particularly relevant in stroke [97]. Lower adiponectin levels in obese individuals are associated with greater stroke damage and increased mortality [98]. Adiponectin protects the brain by enhancing blood–brain barrier integrity, microvascular MMP-9 expression and reducing inflammatory cytokines. Furthermore, adiponectin promotes cerebral blood flow during ischemia by activating endothelial nitric oxide synthase (eNOS) [99]. It also reduces pro-inflammatory cytokines, likely by inhibiting NF-κB through its receptors [98]. Obesity, with its associated decrease in adiponectin, impairs these protective effects, contributing to greater stroke damage and complications. While leptin, another adipokine, has mixed effects on stroke outcomes, its role is less clear and seems to vary based on factors like dose and type of ischemia [100].

In obese mice, stroke triggers a distinct immune response characterized by enhanced neutrophil infiltration into the brain, particularly in areas with significant ischemic damage. This is linked to increased brain chemokine expression (CXCL-1 and CCL3) and higher circulating neutrophil levels, contributing to worsened stroke outcomes. Unlike control mice, obese mice do not exhibit the same neutrophil migration to the liver after stroke, which correlates with a lack of CXCL-1 expression in the liver. This altered inflammatory response suggests that obesity intensifies central brain inflammation while diminishing peripheral inflammatory responses, such as in the liver [101].

BBB research has evolved with the concept of the neurovascular unit (NVU), which encompasses not only endothelial cells but also astrocytes, pericytes, neurons, and other components. This unit highlights the BBB’s crucial role in maintaining CNS homeostasis and supporting normal neuronal function through its interactions with various CNS cell types and peripheral blood-borne factors. BBB dysfunction, characterized by the loss of structural integrity and normal function, is a significant pathological feature in numerous neurological disorders, including stroke [102].

Obesity significantly exacerbates blood–brain barrier (BBB) dysfunction through inflammatory and metabolic disturbances. The chronic low-grade inflammation associated with obesity leads to elevated levels of pro-inflammatory cytokines such as tumor necrosis factor-alpha (TNF-α), interleukin-6 (IL-6), and interleukin-1 beta (IL-1β) [103]. These cytokines impair BBB integrity by increasing its permeability and disrupting endothelial cell function. Additionally, obesity-induced changes in lipid and glucose metabolism further compromise BBB stability, leading to increased endothelial cell damage. Consequently, this disruption facilitates the infiltration of harmful substances into the brain, promoting neuroinflammation and increasing the risk of neurological disorders. Thus, obesity not only impairs BBB function but also amplifies the potential for neurovascular and cognitive complications [104].

### Monocytes and post stroke hematopoiesis: m1/m2 phenotypes

Ischemic stroke triggers a cascade of inflammatory responses involving the activation and recruitment of immune cells, particularly monocytes. Following the initial brain injury, resident microglia are rapidly activated within minutes, releasing signals that recruit circulating immune cells, including monocytes, to the site of injury [105].

## Acute phase: microglial activation and monocyte recruitment

In the immediate aftermath of the stroke, damage-associated molecular patterns (DAMPs) released by dying cells stimulate an emergency hematopoietic response in the bone marrow. This response leads to the increased production and mobilization of monocytes [106]. These monocytes, originating from the bone marrow, are equipped with various receptors, such as toll-like receptors and chemokine receptors like CX3CR1 and CCR2, which facilitate their recruitment to the brain. They are characterized by specific clusters of differentiation, such as CD115, CD11c, CD14, and CD16 in humans, or CD115, CD11b, and Ly6C in rodents [107, 108]. Initially, microglia adopt an anti-inflammatory M2 phenotype, which promotes tissue repair and immune regulation [109].

## Subacute phase: monocyte differentiation and macrophage activation

As the inflammatory response progresses, monocyte-derived macrophages (MDMs) become more prominent, especially 3–7 days after the stroke, marking the transition to the chronic phase of the injury [110]. During this period, microglia and macrophages exhibit a dynamic range of activation states. While they initially adopt the M2 phenotype to support tissue healing, many begin to switch to the pro-inflammatory M1 phenotype. This shift can exacerbate inflammation, potentially worsening brain damage [111]. However, some studies indicate that Ly6Chi (CCR2 +) macrophages, initially pro-inflammatory, can transition back to an anti-inflammatory state at the injury site within 48 h, aiding in the resolution of inflammation by releasing factors like vascular endothelial growth factor and TGF-β [112].

## Chronic phase: neurogenesis and functional recovery

In the later stages, activated microglia and macrophages play a crucial role in neurogenesis and tissue repair [113]. They support the migration and differentiation of neuroblasts from neurogenic regions of the brain, such as the hippocampus and subventricular zone, into new neurons that integrate into damaged circuits [114, 115]. Additionally, these cells produce trophic factors that stimulate axonal regrowth and regeneration, facilitating functional recovery [116]. However, the dual role of microglia and macrophages can be complex; while they are essential for recovery, their activation can also lead to the release of cytotoxic factors, such as reactive oxygen species and TNF, that may worsen ischemic damage and inflammation if not properly regulated [117].

Monocytes and their derived macrophages exhibit a complex and multifaceted role in ischemic stroke recovery. Initially crucial for stabilizing the injury and promoting repair, their shifting phenotypes from M2 to M1 can either aid in recovery or exacerbate damage depending on the timing and context of their activation. Understanding the balance between these phenotypes is key to developing targeted therapies that could improve outcomes following a stroke [105, 106, 109, 111, 113, 117].

### Post stroke rehabilitation, role of aging and obesity

One of the main causes of disability and a lower quality of life is stroke. Therefore, improving the functional and cognitive abilities of patients with stroke is the aim of post-stroke rehabilitation, a multidisciplinary and difficult process that is especially significant for elderly people, who experience particular challenges as a result of aging and obesity.

## Role of aging in post stroke rehabilitation

The risk of stroke increases significantly with age. More than 50% of all strokes occur in individuals over the age of 75, and 30% occur in those over 85. In aging societies, particularly in the Western world, there is a noticeable rise in stroke incidence around the age of 73. Although advances in stroke medicine and improved management of risk factors have contributed to reduced incidence and increased survival rates, the overall burden of stroke has surged again due to population aging, growing obesity rates, and the rising prevalence of metabolic syndrome [118, 119].

Older adults not only face a higher risk of stroke but also experience worse outcomes. Stroke in the elderly is associated with higher mortality, prolonged hospitalizations, lower functional recovery, and a greater likelihood of institutionalization. Recovery after stroke is generally poorer with advancing age, prompting recommendations that rehabilitation intensity be adjusted according to age and stroke severity. However, it is often difficult to disentangle the effects of aging itself from age-associated comorbidities—such as cardiovascular disease, hypertension, diabetes, and cognitive impairment—which independently contribute to diminished recovery potential [120].

## Age-related changes in brain plasticity

Aging also affects brain plasticity, the brain’s capacity to reorganize its structure and function in response to internal and external stimuli. While neuroplasticity persists throughout life, its mechanisms shift with age. In younger brains, plasticity is largely driven by dynamic and competitive processes, whereas in older brains, it becomes more selective and outcome-dependent [121].

One molecular hallmark of this decline is the age-related reduction in synaptophysin, a key synaptic vesicle protein. Lower levels of synaptophysin weaken the ability of synapses to adapt and remodel, contributing to cognitive decline and reducing the brain’s capacity to recover from injury, such as stroke. This diminished synaptic plasticity undercuts both neurorehabilitation and cognitive resilience in older adults [122].

Successful stroke recovery also depends on the brain’s ability to repair itself, notably through restoration of the blood–brain barrier (BBB). Aging compromises BBB integrity through epigenetic mechanisms, including widespread alterations in DNA methylation. Genes involved in neuroprotection and vascular health—such as the estrogen receptor, IGF2, and p16—undergo hypermethylation with age, leading to long-lasting functional changes [123]. Further, aging shifts the BBB’s DNA methylome and transcriptome, particularly in genes related to angiogenesis and structural maintenance. Although aging endothelial cells retain some ability to promote new vessel growth, they show epigenetic changes that inhibit effective repair. Specifically, hypomethylation and overexpression of *Foxo1*, an angiogenic repressor, alongside decreased activity of angiogenic regulators such as *Klf8* and *Epha6*, impair vascular regeneration [116–124]. In addition, the hypermethylation and reduced expression of *claudin-5*, a crucial tight junction protein, further compromise BBB repair capacity [125].

## Role of obesity in post-stroke rehabilitation

Obesity is a major global health concern and a well-established independent risk factor for stroke. It contributes to numerous stroke-related comorbidities, including metabolic syndrome, hypertension, diabetes, and hypercholesterolemia, all of which negatively influence stroke incidence and outcomes. Clinically, obese individuals often experience more severe strokes and poorer recovery trajectories.

Experimental studies in obese rodents have confirmed these observations, showing increased ischemic damage, compromised blood–brain barrier (BBB) integrity, and a greater risk of hemorrhagic transformation following stroke, all of which contribute to worse neurological outcomes [126]. One of the key mechanisms linking obesity to poorer stroke outcomes is chronic systemic inflammation. Obesity is characterized by a persistent low-grade inflammatory state that not only exacerbates the initial brain injury but also impairs the resolution of post-stroke inflammation, thereby hindering tissue repair and recovery processes [127]. This link between obesity and heightened cerebral inflammation after stroke has been further demonstrated in preclinical models. Obese mice fed a high-fat diet (60%) displayed significantly larger infarct volumes following stroke, accompanied by increased BBB permeability, elevated levels of chemokines such as CXCL-1 and CCL3, and greater infiltration of immune cells, including neutrophils and activated microglia [101].

At the molecular level, obesity and associated atherosclerosis promote microglial activation and neuroinflammation. PET imaging studies reveal increased microgliosis in obese subjects compared to lean controls. Additionally, in atherosclerotic *ApoE-/-* mice on a high-fat diet, there is upregulation of vascular adhesion molecules such as ICAM and VCAM. These changes are associated with increased infiltration of CD45⁺ leukocytes—primarily granulocytes—into the choroid plexus, further contributing to neuroinflammation and potential disruption of cerebrospinal fluid homeostasis [128].

### Treatments for post-stroke systemic inflammation in animal models and patients

Stroke remains a leading cause of death and long-term disability worldwide, placing a significant burden on public health and socioeconomic systems [129]. Current therapeutic approaches for ischemic stroke primarily focus on acute revascularization through thrombolytic agents or endovascular therapy to restore cerebral blood flow. However, these treatments are constrained by narrow therapeutic windows and various contraindications, limiting their application [130]. Consequently, there is a critical need for novel treatment strategies. Systemic inflammation following stroke has emerged as a promising research target, with clinical trials exploring ways to mitigate inflammation to improve patient outcomes and prevent secondary complications [84].

## Anti-inflammatory therapies in animal models

Animal models of ischemic stroke have been crucial in exploring the effects of anti-inflammatory therapies. These models allow for controlled experimentation on the timing, dosage, and mechanisms of action of potential treatments, providing insights that inform clinical trials [131].

Minocycline, a tetracycline antibiotic, has gained attention for its anti-inflammatory, antioxidant, and anti-apoptotic properties. Preclinical studies demonstrate that minocycline can reduce ischemic infarct volume and neurological deficits post-stroke. At the molecular level, minocycline lowers TNFα levels and increases HSP70 and HuR protein levels in the penumbra. This shift in HuR binding preference towards HSP70 post-ischemia suggests a protective response, correlating with improved motor performance and highlighting minocycline’s potential as a clinical therapy [132, 133].

Tumor necrosis factor (TNF)-α is released in the brain after ischemic stroke, contributing to neuronal apoptosis and playing a dual role in stroke pathology, as it can both promote inflammation that worsens stroke progression and contribute to cerebral tolerance against hypoxia and ischemia [134, 135]. Traditional TNF inhibitors, such as etanercept, do not cross the blood–brain barrier (BBB). To address this, researchers have developed a BBB-penetrating TNF inhibitor by fusing a type II human TNF receptor with a chimeric monoclonal antibody against the mouse transferrin receptor. This fusion protein, acting as a “molecular Trojan horse,” significantly reduced stroke volumes and neural deficits in mice, showing its potential as a neuroprotective treatment [136].

Interleukin-1 (IL-1) is produced by monocytes, glia, neurons, and endothelial cells, and exacerbates stroke outcomes through mechanisms such as leukocytosis, brain edema, and blood–brain barrier breakdown. IL-1 receptor antagonist (IL-1Ra), a human-derived polypeptide, blocks IL-1 by binding to its receptors. Experimental studies with rats having permanent middle cerebral artery occlusion demonstrated that IL-1Ra significantly reduced necrotic neurons, improved neurological scores, and decreased PMN leukocytes in the ischemic hemisphere, indicating its potential to improve outcomes in preclinical stroke models [137].

Statins, specifically 3-hydroxy-3-methylglutaryl coenzyme A (HMG-CoA) reductase inhibitors, have shown promise in reducing infarct volumes and stroke severity in animal models. Beyond their lipid-lowering effects, statins exhibit pleiotropic benefits, including promoting angiogenesis, enhancing endothelial nitric oxide synthase activity, improving cerebral blood flow, supporting neural repair, and possessing anti-inflammatory and antioxidant properties. Observational studies and meta-analyses suggest that administering statins around the time of stroke can lead to improved functional outcomes and reduced mortality [138]. Statins also help by suppressing activated microglial cells, reducing reticulum stress through autophagy inhibition, and decreasing brain edema by inhibiting aquaporin 4 (AQP4) expression [139].

Exosomes, a type of extracellular vesicle (EV), play a significant role in post-stroke recovery by facilitating intercellular communication and modulating inflammation [140]. Derived from cells like neurons and mesenchymal stromal cells (MSCs), exosomes transfer proteins, lipids, and genetic material, including microRNAs (miRNAs), across the neurovascular unit [141]. In animal models, MSC-derived exosomes, particularly those obtained from hypoxic conditions, have shown potential to enhance angiogenesis, reduce neuroinflammation, and support blood–brain barrier integrity after stroke [142]. Additionally, exosomal miRNAs like miR-21 and miR-124 contribute to neuroprotection, neural plasticity, and the prediction of functional recovery in stroke patients [143].

## Clinical trials and therapeutic approaches in patients

Clinical studies are critical for advancing our understanding and treatment of post-stroke inflammation, significantly impacting stroke outcomes. Research underscores the role of inflammation in exacerbating brain injury and influencing recovery, highlighting the need for targeted therapies. Ongoing clinical research is vital for validating these therapies and optimizing treatment strategies to enhance overall stroke management [144, 145].

Minocycline, known for its anti-inflammatory and protease-inhibiting properties, has demonstrated promise in preclinical stroke models [146]. Clinical trials have reported that adverse effects of minocycline are generally mild and unrelated to dosage. With a strong safety record from extensive use, minocycline significantly improves stroke outcomes when administered orally for 5 days after acute ischemic stroke. As an adjunct to tissue plasminogen activator (tPA), minocycline does not interfere with tPA’s fibrinolytic effect and helps reduce reperfusion hemorrhage. Intravenous minocycline, administered at doses of 3 to 10 mg/kg daily for 3 days, achieves neuroprotective serum concentrations in experimental models [147].

Pro-inflammatory cytokines, such as interleukin-1 (IL-1), worsen stroke outcomes. A systematic review evaluating the IL-1 receptor antagonist (IL-1Ra), known as anakinra, found that it generally leads to better outcomes in terms of biomarkers, clinical assessments, and adverse events. Administered within three hours of stroke onset, IL-1Ra has shown promise as a neuroprotective treatment, offering potential therapeutic benefits for acute cerebrovascular disease [148].

Experimental data also highlight the role of inflammatory cells throughout atherosclerosis development. This process begins with the transendothelial migration of circulating monocytes, triggered by adhesion molecules responding to factors such as arterial tension, non-laminar flow, cigarette smoke, and angiotensin 2. Once in the arterial wall, low-density lipoprotein (LDL) lipids become trapped, leading to the differentiation of monocytes into lipid-laden ‘foamy’ macrophages [149, 150].

The Stroke Prevention by Aggressive Reduction of Cholesterol (SPARCL) study demonstrated that statins effectively reduce the incidence of strokes and cardiovascular events in patients with stroke or transient ischemic attack, primarily through their cholesterol-lowering properties. However, the study also noted a slight increase in hemorrhagic stroke incidence among patients using statins [144].

## Conclusions and future perspectives

**Fig. 2:**
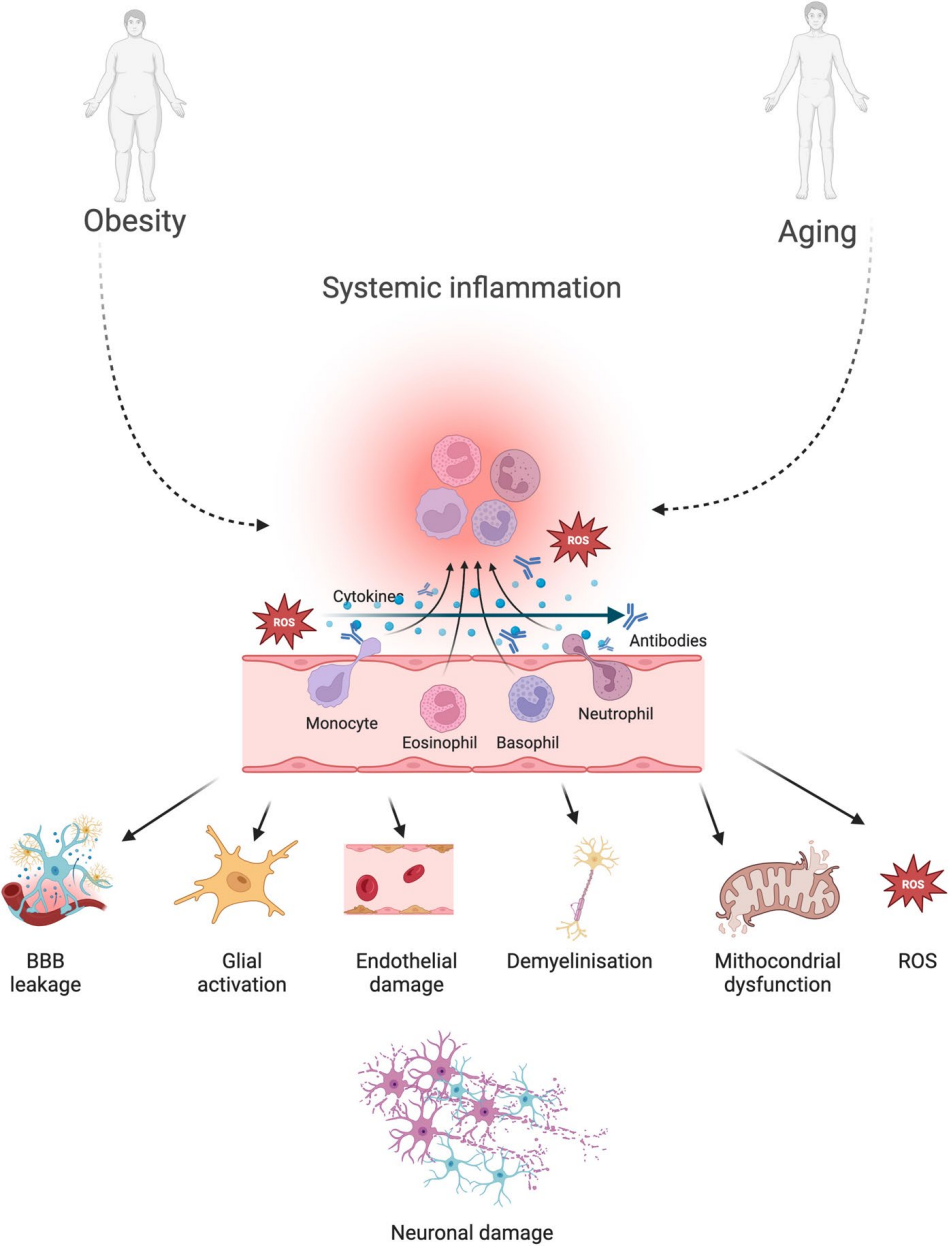
Obesity- and aging-driven systemic inflammation as a convergent pathway to neurovascular and neuronal injury. Both obesity and aging contribute to a state of chronic systemic inflammation characterized by elevated cytokines, reactive oxygen species (ROS), circulating antibodies, and activated immune cells (monocytes, eosinophils, basophils, neutrophils). These inflammatory mediators disrupt neurovascular homeostasis through multiple mechanisms, including increased blood–brain barrier (BBB) permeability, glial activation, endothelial injury, demyelination, and mitochondrial dysfunction. The resulting oxidative stress and immune dysregulation amplify neuronal vulnerability, ultimately leading to progressive neuronal damage. This figure highlights systemic inflammation as a shared mechanistic axis linking obesity and aging to neurodegeneration

Systemic inflammation following ischemic stroke arises from a complex interplay between pro-inflammatory cytokines, immune cells, and neurovascular mechanisms. Both aging and obesity amplify these responses, worsening stroke severity and impeding recovery (Fig. 2). Aging induces a chronic, low-grade inflammatory state—*inflammaging*—that exacerbates neuronal injury, impairs plasticity, and delays functional repair [98, 102, 151]. Obesity, characterized by metabolic dysregulation and a persistent proinflammatory milieu, further compounds these effects by disrupting immune and vascular homeostasis, resulting in larger infarct volumes and poorer rehabilitation outcomes [106, 107, 109]. The synergistic impact of aging and obesity on systemic inflammation thus represents a major challenge to post-stroke recovery and long-term brain health.

Future research should address several critical directions to improve prevention and treatment strategies. First, there is a need to develop targeted anti-inflammatory therapies capable of modulating systemic and neurovascular inflammation without inducing immunosuppression. Agents such as minocycline, beyond its antimicrobial activity, have shown promise in preclinical and early clinical studies by reducing infarct volume, dampening cytokine release, and improving functional recovery [132, 146]. Larger, well-controlled trials are warranted to confirm its efficacy and safety in human stroke populations.

Second, a deeper understanding of the molecular and cellular mechanisms that link aging, obesity, and systemic inflammation may yield novel therapeutic targets. Investigating adipokines such as adiponectin and their influence on endothelial and blood–brain barrier integrity may clarify how metabolic inflammation drives neurovascular dysfunction [94, 109, 110]. Similarly, dissecting the phenotypic polarization of monocytes and macrophages during stroke recovery could identify immunomodulatory strategies that restore protective immune balance [106, 107, 120, 122].

Third, personalized and time-sensitive therapeutic strategies should account for individual risk factors such as age, obesity, and biological characteristics—including circadian variations in inflammatory activity and stem cell responsiveness. Incorporating these variables into treatment design could optimize rehabilitation protocols and pharmacological interventions [129, 130, 150, 152]. Given the current limitations of acute stroke therapies, prioritizing prevention remains paramount. Identifying individuals at elevated risk through molecular, metabolic, and lifestyle profiling will enable earlier intervention and more effective control of modifiable risk factors.

Fourth, as underscored in the emerging paradigm of multimodal and stratified therapy, no single agent is likely to address the multifaceted nature of stroke pathology. Integrating anti-inflammatory, vascular-protective, and metabolic interventions offers a pragmatic route forward. Pharmacologic agents targeting the IL-1 pathway or minocycline can attenuate neuroinflammation, while statins and renin–angiotensin system modulators (ACE inhibitors or ARBs) support endothelial stability and cerebral perfusion [153–155]. The INSPIRES randomized clinical trial [156] demonstrated that immediate intensive statin therapy after mild ischemic stroke or high-risk TIA significantly reduced early recurrent ischemic events compared with delayed initiation. Complementary metabolic optimization through GLP-1 receptor agonists and SGLT2 inhibitors, as shown in trials such as DURATION-8 [157, 158], improves glycemic control, vascular function, and systemic inflammation, collectively lowering cardiovascular and cerebrovascular risk [160,161].

Beyond pharmacology, structured exercise and nutritional interventions remain potent modulators of cytokine tone, insulin sensitivity, and neurovascular resilience. Together, these integrated interventions could mitigate inflammaging, restore endothelial integrity, and reduce recurrent stroke risk in older, obese, and multimorbid patients.

In summary, targeting systemic inflammation through mechanistically informed, personalized, and multimodal strategies holds substantial promise for improving stroke outcomes. Future work should bridge molecular insights with clinical implementation, through phenotypically stratified trials and prevention-first frameworks, to advance precision cerebrovascular medicine and enhance the quality of life for stroke survivors. 

## Data Availability

No new datasets were generated during the current study.
